# Early propranolol treatment of infantile hemangiomas improves outcome^☆^

**DOI:** 10.1016/j.abd.2022.04.008

**Published:** 2022-12-26

**Authors:** Ana Giachetti, María Sol Díaz, Paula Boggio, María Lourdes Posadas Martínez

**Affiliations:** a Pediatric Dermatology Section, Pediatric Department, Hospital Italiano de Buenos Aires, Buenos Aires, Argentina; b Internal Medicine Research Area, Research Department, Hospital Italiano de Buenos Aires, Buenos Aires, Argentina

**Keywords:** Child, Hemangioma, Propranolol, Propanolol/therapeutic use

## Abstract

**Background:**

Infantile hemangiomas (IH) are the most common soft tissue tumors of childhood. Although most of these tumors are not worrisome, some IH may be life or function-threatening, can lead to permanent disfigurement, or have associated structural congenital anomalies, requiring early recognition and referral to specialists for treatment consideration. Since 2008, oral propranolol has been widely considered to be the first-line treatment for IH.

**Objectives:**

To evaluate aesthetic and functional outcome in propranolol-treated infantile hemangiomas according to the age of treatment onset.

**Methods:**

Retrospective, observational study of infantile hemangioma patients under 4 years of age at the time of diagnosis, treated with oral propranolol. Evaluated parameters included: pre and post-treatment morphologic/aesthetic aspects of the hemangioma, total resolution rate, degree of functional compromise of affected areas and its evolution. Two independent pediatric dermatologists evaluated all cases reviewing clinical data from medical records and comparing clinical photographs taken at initiation and at the end of treatment of each patient. Data were analyzed with STATA 13.0 program.

**Results:**

The cohort included 138 patients, with a female predominance. The median age at therapy onset was 3 months. The morphological/aesthetic improvement rate was 99% (95% CI 96‒99), the total resolution rate was 48% (95% CI 44‒60) and the functional improvement rate reached 100%. When comparing total resolution outcome versus age when treatment started, the improvement was larger in younger patients (3.5 vs. 4.9 months, p = 0.01). When comparing the total resolution rate in those younger or older than 3 months at treatment initiation, the percentage of total resolution in the younger group was 57% vs. 40% in the older one (p = 0.05).

**Study limitations:**

Retrospective design; patients photographs were the sole indicators used to measure regression rates. Visual assessment is subjective.

**Conclusion:**

The present results strongly suggest that early (before 3 months of age) initiation of treatment of infantile hemangiomas with propranolol results in significantly higher aesthetic and functional improvement rates and a higher percentage of total resolution.

## Introduction

According to the International Society for the Study of Vascular Anomalies (ISSVA), vascular lesions are classified into two major types: tumors and vascular malformations.^1^ Likewise, tumors are divided into 3 large groups: benign, borderline and malignant. Infantile hemangiomas belong to the group of benign tumors, and they are the most common soft tissue tumors of childhood, with an estimated incidence of 3% to 10%.^2^ Lesions are typically absent at birth, although in hardly 50% of cases a subtle precursor such as a telangiectatic macule surrounded by a pale halo, an erythematous or pale macule, or less commonly a bruise or scratch-like lesion can be found.^3^

The natural history of IH is characterized by a rapid proliferative phase that occurs typically within the first months of life, followed by slow spontaneous involution, that may last until the age of 4 years.^4^, ^5^ Although most of these tumors are not worrisome, some IH may be life or function-threatening, can lead to permanent disfigurement or have associated structural congenital anomalies, requiring early recognition and referral to specialists for treatment consideration (Table 1).^6^

**Table 1:** High-risk IH.

According to IH anatomic location	Associated risk
Face	Aesthetic sequel
Nasal tip	Aesthetic sequel, ulceration and scarring
Lips or perioral	Aesthetic sequel, ulceration, scarring and feeding difficulties
Periocular	Aesthetic sequel and eye functional involvement
Ear	Aesthetic sequel and ear functional involvement
Parotid	Functional involvement and facial nerve compression
Beard area	Airway IH
Mammary	Aesthetic sequel
Folds (perineum, axilla, neck)	Ulceration and scarring
**According to IH morphology**	
Focal with stepped border	Aesthetic sequel and scarring
Segmentary	Associated malformations, aesthetic sequel, ulceration and scarring
**According to IH distribution**	
Segmentary on the head and neck	PHACE syndrome
Segmentary on the lumbosacral area	LUMBAR syndrome
**According to IH number**	
Multiple hemangiomas (≥5)	Visceral involvement

Medical systemic therapy is typically indicated in 1) Life-threatening IH; 2) IH with functional involvement; 3) Ulcerated HI; 4) IH with risk of permanent scarring or disfigurement.^3^, ^7^ Since 2008, oral propranolol has been widely considered to be the first-line treatment for IH.^8^

Herein, the authors report a series of 138 patients with HIs treated with oral propranolol and objectively demonstrate that early treatment improves outcomes.

## Methods

A retrospective, observational study was conducted at the Pediatric Dermatology Section of the Hospital Italiano de Buenos Aires, Argentina, between January 2009 and December 2019. The study included IH patients under 4 years of age at the time of diagnosis, treated with oral propranolol. Prior to the treatment, a cardiologic evaluation was performed. All patients were treated in an outpatient setting, with the exception of premature, low-weight infants and patients with comorbidity that started treatment while admitted to the hospital.

Patients received 1 to 3 mg/kg/day of oral propranolol, divided into two or three doses until no further improvement was observed, which occurred on average at 12.5 months of treatment. Outcomes evaluated in this study included: Morphologic/aesthetic aspect of the IH pre- and post-treatment, including color intensity, thickness, presence of deep components, distortion of local anatomic reference points, ulceration, and residual scarring. The total resolution, is defined as complete involution of the hemangioma or persistence of few residual telangiectasias or minimal skin texture alterations. Functional compromise of affected areas, from absent to maximum.

The analysis was performed by two independent pediatric dermatologists who evaluated all cases by reviewing clinical data from medical records and comparing clinical photographs taken at initiation and at the end of each patient treatment.

Table 2, created by the authors, shows the numerical scales used to evaluate each of these aspects and create a morphologic/aesthetic improvement numeric value for each patient. Patients with total values between 5 and 8 were interpreted as displaying high improvement, those with values between 9 and 12 were considered as displaying medium improvement and patients with values between 13 and 17 were interpreted as displaying poor improvement.

**Table 2:** Scales used to evaluate the main aspects of IH.

Morphologic/Aesthetic aspect				
**a) Color intensity (5-points)**				
1) Light pink (barely perceptible)	2) Pink	3) Mottled red	4) Dull red	5) Bright red
**b) Tumor thickness (3-points)**				
1) Flat	2) Slightly raised	3) Strongly raised		
**c) Deep component (3-points)**				
1) Absent	2) Mild	3) Moderate to severe		
**d) Distortion of local anatomic reference points (4-points)**				
1) Absent	2) Mild	3) Moderate	4) Severe	
**e) Ulceration and residual scarring**				
1) Absent	2) Present			
**Total resolution**				
Yes	No (incomplete involution determined for persistence of few residual telangiectasias or minimal skin texture alterations)			
**Functional compromise of affected areas**				
1) Absent	2) Mild	3) Moderate	4) Severe	

Data were analyzed with STATA 13.0 program.

Ethical approval was obtained from the Research Protocol Ethics Committee of the Hospital Italiano de Buenos Aires on February 28^th^, 2019 (protocol number 4058).

## Results

The cohort included 138 Caucasian patients, 107 girls, and 31 boys, and a total of 145 HIs.

The most frequent localization of IH was head and neck (92 lesions, 66.6%) followed by perineal area (18 lesions, 13%), extremities (17 lesions, 12.3%), trunk (15 lesions, 10.8%) and hepatic (3 lesions, 2.2%). According to distribution, 113 patients had focal IH, 13 multifocal IH, and 12 segmentary IH, among them 3 cases corresponded to PHACE syndrome and 1 to PELVIS syndrome. Based on their depth, the authors found 53 superficial IH, 37 deep IH, and 55 combined.

All patients received oral propranolol and in cases where ulceration developed, the authors added topical treatment (petrolatum alone and ointments with metronidazole or mupirocin) and oral paracetamol. No other systemic treatment was associated with propranolol in this series.

Treatment was initiated at a mean age of 3 months, with patients, started on propranolol as early as 1 month, and as late as 18 months. Sixty-five patients started treatment before 3 months of age, and the remaining 73 after that time.

**Figure 1:**
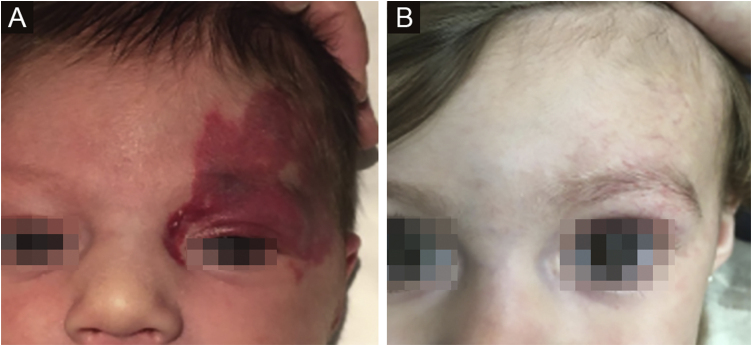
(A) Pre-treatment initiation at 3 weeks old. (B) Post-treatment, at 12 months old (patient # 50).

**Figure 2:**
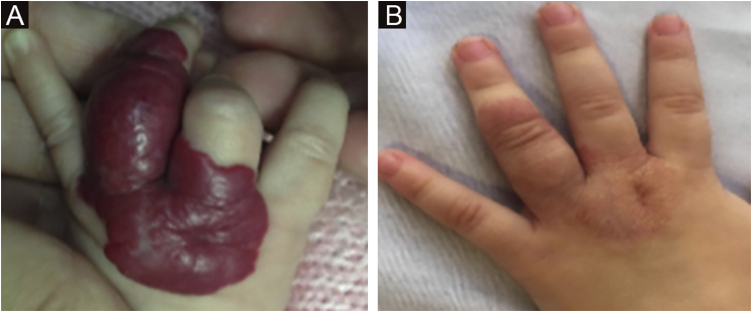
(A) Pre-treatment initiation at 8 weeks old. (B) Post-treatment, at 20 months old (Patient #98).

**Figure 3:**
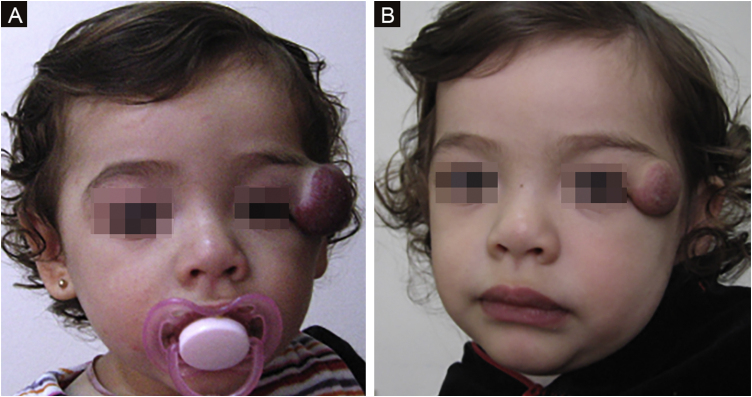
(A) Started treatment at 9 months old. (B) Poor response at 20 months old, when propranolol was stopped. Required surgical management (Patient #1).

The morphological/aesthetic improvement seen in the patients, using the numerical scale shown in Table 2, indicated that all of them presented pretreatment values between 6 and 15 (median = 9, IR 8‒11). High improvement was observed in 49 patients for a rate of 35.5%, medium improvement was observed in 84 patients, for a rate of 60.9%, and 5 patients (4.3%) did not show amelioration. Figure 1, Figure 2 show treatment response in two patients displaying high morphological/aesthetic improvement and Fig. 3 illustrates a case with a poor improvement that required complementary surgical treatment.

Propranolol treatment was discontinued at a mean age of 12.5 months, until no further improvements were observed, with patients finishing as early as 9 months, and as late as 36 months.

The total resolution rate of IH observed in this cohort was 48% (n = 66, 95% CI 44%‒60%); and the full functional improvement rate was 100% (n = 138).

When comparing total resolution outcome versus age when treatment started, the improvement was larger in younger patients (3.5 vs. 4.9 months, p = 0.01). When comparing the percentage of total resolution in those younger or older than 3 months at treatment initiation, the younger group showed a total resolution of 57% vs. 40% in the older one (p = 0.05).

## Discussion

Although most IH does not require intervention, there is a subset of them that need prompt treatment to prevent complications. The pediatrician has a crucial role in identifying those lesions that will require intervention and in referring the patient to the pediatric dermatologist. Considering that the period of accelerated growth of IH occurs between 5.5 and 7.5 weeks of life, reference to the specialist should ideally be carried out at that time^7^ seeking the window of opportunity for optimal treatment.^4^

Propranolol is a non-selective beta blocker that is considered the current treatment of choice for IH that require systemic therapy.^8^ The most commonly used doses of propranolol are 1 to 1.5 mg/kg twice daily. A recommendation is to start with 1 mg/kg/day and increase it over a month until reaching the target dose.^9^

There is still no consensus regarding the duration of treatment, but therapy until at least 12 months of age is desirable, to cover the proliferative phase and reduce the risk of relapse.^10^ The patients in this cohort were medicated on average for 12.5 months until no further improvements were observed.

A study in which family photographs were evaluated concluded that the faster IH growth period occurs before 8 weeks of age and that the optimal time for a referral or for treatment initiation was 1 month of age, far earlier than the time most patients are referred to the specialists.^5^ In this series, when comparing total resolution outcome versus age when treatment started, the improvement was larger in younger patients (3.5 vs. 4.9 months, p = 0.01) and the percentage of a total resolution was also greater in those younger than 3 months at treatment initiation (57% vs. 40%, p = 0.05). Achieving these positive results could prevent serious functional losses such as the potential loss of vision in a periocular hemangioma or difficulty feeding in an ulcerated hemangioma on the lips. It may also prevent serious cosmetic sequelae that may require surgical interventions. Therefore, for those IH requiring treatment, prompt initiation decreases morbidity and improves long-term outcomes.

Propranolol has an excellent safety profile and high tolerability.^11^ The most commonly reported adverse reactions are the coolness of the distal extremities and sleep disturbances which can improve with a reduction in dosage or by giving the evening dosing earlier, respectively.^5^, ^10^ However, the most serious potential complication is hypoglycemia, which can be prevented by administering the medication after feeding and stopping it during illnesses in which the child is not feeding adequately. Other side effects are gastrointestinal symptoms and bronchial hyperreactivity which may require temporary discontinuation of treatment. Even though bradycardia and hypotension are very infrequent in healthy children and when they occur are usually asymptomatic, guidelines suggest controlling the heart rate and arterial pressure 2 hours after the first dose of propranolol and also after an increase of the dose.

Nowadays, it is recommended to perform an ECG screening only in infants with a baseline heart rate below normal for age, or in those with a family history of congenital heart conditions, arrhythmias, or a maternal history of connective tissue disease.^10^

Patients with PHACE syndrome require special consideration because they have an increased risk of having cardiac and cerebrovascular anomalies as well as stroke; so it is preferable to use the lowest effective dose of propranolol, divided into three daily doses, in order to prevent large fluctuations in blood pressure.^12^

## Conclusion

Early intervention is critical in treating IH. This study demonstrates that the earlier propranolol treatment starts, the best outcome is obtained. The present results emphasize that early initiation of treatment of IH with propranolol, mainly before 3 months of age, results in significantly higher aesthetic and functional improvement rates as well as a higher percentage of total resolution.

The role of the pediatrician is essential in recognizing IH which may require treatment, and referring these patients promptly to the pediatric dermatologist, in order to start treatment early and obtain the maximum benefit from it.

## Financial support

This research received no specific grant from any funding agency in the public, commercial or not-for-profit sectors.

The authors declare that they have no known competing financial interests or personal relationships that could have appeared to influence the work reported in this paper.

## Authors’ contributions

Ana Giachetti had the idea and initiated the research. Ana Giachetti and Maria Sol Diaz collected, analyzed and interpreted the data. Lourdes Martinez Posadas did the statistical analysis. Paula Boggio and Maria Sol Diaz participated in writing the paper and critically reviewing the intellectual content. All authors revised and approved the final manuscript.

## Conflict of interest

None declared.
